# (Poly)phenol-gut microbiota interactions and their impact on human health

**DOI:** 10.1097/MCO.0000000000001132

**Published:** 2025-04-28

**Authors:** Xiaohe Liu, Amnah Alharbi, Rachel Gibson, Ana Rodriguez-Mateos

**Affiliations:** a Department of Nutritional Sciences, School of Life Course and Population Sciences, Faculty of Life Sciences and Medicine, King's College London, London, UK; b Department of Biochemistry, Faculty of Science, University of Tabuk, Tabuk, Saudi Arabia

**Keywords:** (poly)phenols, gut microbiota, health effects, metabolism, microbial metabolites

## Abstract

**Purpose of review:**

(Poly)phenols are bioactive compounds in plant-based foods with well documented health benefits. Their metabolism in dependent on gut microbiota composition, diversity, and function, which modulate their bioavailability and physiological effects. This review examines recent insights into (poly)phenol-gut microbiota interactions, emphasizing their relevance in mediating health benefits and mechanisms of action.

**Recent findings:**

Recent studies have identified a high interindividual variability in (poly)phenol metabolism, explored microbial-derived metabolites with potential health benefits, and revealed bidirectional influences between (poly)phenols and gut microbiota composition. Advances in analytical techniques and large-scale studies have refined our understanding of these interactions, but inconsistencies remain in linking specific microbial signatures to health outcomes.

**Summary:**

Standardized methodologies and long-term studies are needed to clarify the impact of (poly)phenol-derived metabolites on human health. Future research should focus on personalized and targeted nutrition approaches to optimize (poly)phenol benefits.

## INTRODUCTION

(Poly)phenols are a diverse group of naturally occurring phytochemicals abundant in plant-based foods, including fruits, vegetables, tea, coffee, nuts and cocoa. (Poly)phenols have gained attention due to their health benefits, including anti-inflammatory, neuroprotective, and cardioprotective effects. Epidemiological studies have highlighted (poly)phenols’ protective effects on cardiovascular diseases, type 2 diabetes, and cancer [1^▪^,^2^,3^▪^]. Emerging evidence also highlights their potential role in neuroprotection and in the prevention of cognitive decline [3^▪^,4^▪^]. Additionally, (poly)phenols act as ‘prebiotic-like’ compounds, selectively promoting beneficial gut microbes and supporting microbial balance, which in turn contributes to systemic health [5^▪^]. Understanding these interactions is essential for exploring their broader physiological implications.

The bioavailability of (poly)phenols is a critical factor in determining their biological activity. While a small fraction is absorbed directly in the small intestine, most (poly)phenols reach the colon, where gut microbiota extensively metabolize them into simpler, potentially more bioactive compounds. However, inter-individual differences in gut microbiota composition and functional capacity lead to significant variability in (poly)phenol metabolism, resulting in diverse physiological responses. Individuals exhibit distinct gut microbial metabolic phenotypes, influencing their ability to convert (poly)phenols into their corresponding gut microbial metabolites. This review explores recent advances in understanding the interplay between (poly)phenol metabolism and gut microbiota composition and its implications for human health. 

**Box 1:**
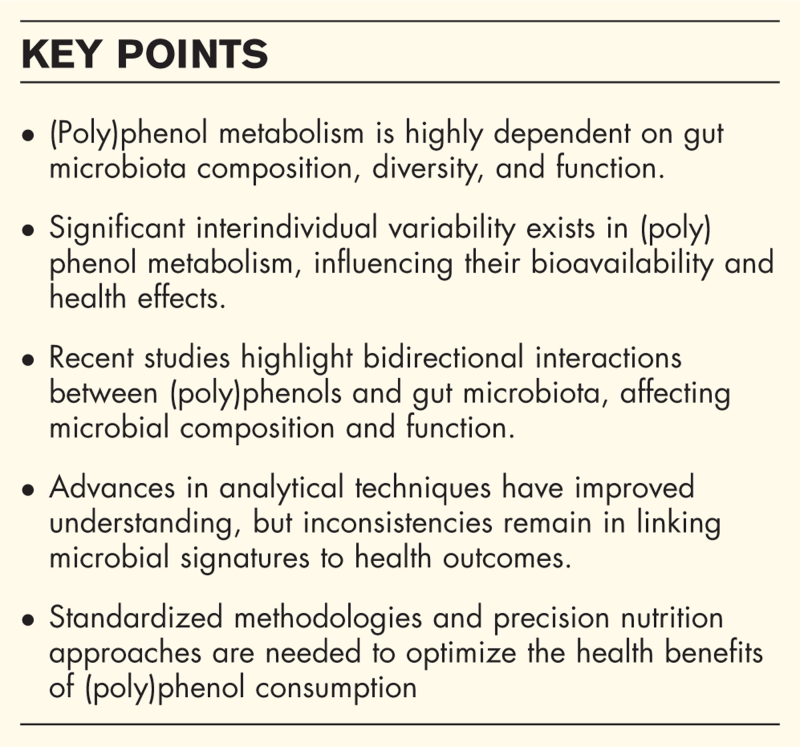
no caption available

## (POLY)PHENOLS AS PREBIOTIC-LIKE MODULATORS OF GUT MICROBIOTA

It is now well established that gut microbiota plays a crucial role in host health, with dysbiosis linked to metabolic and inflammatory disorders [6,7]. Probiotics and prebiotics are well established interventions for modulating gut microbiota composition and promoting health [8–10]. Recently, the definition of prebiotics has expanded beyond carbohydrates to include other nondigestible compounds that are selectively utilized by host microorganisms to confer health benefits [11]. Some novel or emerging prebiotic compounds includes omega-3 fatty acids, resistance dextrin, and amino acids. [12]. This broader classification has positioned (poly)phenols as potential prebiotic-like compounds, capable of selectively modulating microbial communities [5^▪^,^13^,^14^].

Gut microbiota abundance and diversity, key markers of gut health, are modulated by (poly)phenol consumption. The prebiotic-like effects of (poly)phenols primarily occur through selective bacterial fermentation, leading to variable impacts on microbial composition. A recent systematic review on prebiotic modulation of gut microbiota suggested that different (poly)phenols target distinct microbial communities, unlike prebiotics such as inulin and galactooligosaccharides (GOS), which induce more consistent shifts in composition [15^▪^]. This variability arises from structural differences among the diverse (poly)phenol classes, which shape their metabolic pathways. For instance, a recent randomized controlled trial (RCT) found that a three-week supplementation of pomegranate juice (rich in ellagic acid and ellagitannins) increased *Lactobacillus* and *Enterococcus* abundance compared to placebo in overweight and participants with obesity [16^▪^]. A similar study design in athletes reported that 3-week daily supplementation of a dark chocolate drink (rich in cocoa flavan-3-ols) increased the abundance of *Faecalibacterium prausnitzii*, *Ruthenibacterium lactatiformans* and *Flavonifractor plautii*[17].

(Poly)phenols have both antimicrobial and growth promoting effects on the gut microbiota, selectively inhibit pathogenic species while promoting the growth of beneficial bacteria, shaping a healthier microbial ecosystem [18]. This selective pressure enriches taxa capable of metabolizing (poly)phenols into bioactive compounds. A recent cohort validated that consumers with high dietary flavonoid and lignans consumption have more microbial taxa abundance, including *Lactobacillus* and *Sutterella*, compared to low consumers of these (poly)phenol compounds, highlighting the role of these microbial taxa in (poly)phenol metabolism [19]. As for (poly)phenol's bactericidal activities, it is reported that ellagic acid exert antimicrobial effects against bacteria such as *E. coli*[20], contributing to microbiota modulation and anti-inflammatory benefits.

### Gut microbial metabolism of (poly)phenols: challenges in understanding its complexity

Following limited absorption in the small intestine, most (poly)phenols reach the colon, where the gut microbiota mediate extensive biotransformation, generating a diverse range of microbial metabolites. Many metabolic pathways are involved, including A- and C-ring cleavage, C-ring cleavage via dioxygenases, dehydroxylations, and hydrogenations of alkene moieties [21]. However, the precise pathways and key bacteria involved in the formation of specific metabolites remain incompletely understood. Advances in recent years, particularly through metabolomics and microbial sequencing, have improved the identification of key bacterial species and enzymatic reactions involved in (poly)phenol metabolism. For instance, a recent study on a Chinese cohort identified 21 genera positively associated with the capacity to convert isoflavone into equol, which belong to 4 phyla families: Firmicutes, Actinobacteria, Proteobacteria, and Bacteroidetes [22^▪^]. Enterolactone and enterodiol, derived from the bacterial metabolism of plant lignans, are linked to *Lactonifactor longoviformis* and *Ruminococcus* spp. [23^▪^]; Ellagitannins are hydrolysed to ellagic acid, which is further transformed into different urolithins by recently identified strains of *Gordonibacter*, *Ellagibacter* and *Enterocloster* genera [24^▪^].

Despite these advances, several challenges remain. Many bacterial enzymes involved in (poly)phenol metabolism remain unidentified, and their expression may depend on dietary habits, host factors, and microbial interactions. The dynamic nature of the gut microbiota, coupled with the influence of external factors such as antibiotic use and lifestyle, further complicates the predictability of (poly)phenol metabolism. Additionally, the high interindividual variability in gut microbiota composition significantly affects (poly)phenol metabolism, making it difficult to establish generalized metabolic pathways. Addressing these challenges requires integrated approaches combining multiomics technologies, computational modelling, and targeted microbial culturing to fully elucidate the complex pathway of gut microbial transformations of (poly)phenols.

## (POLY)PHENOL METABOTYPES: HYPE OR HOPE IN EXPLAINING VARIABILITY IN RESPONSE?

The significant interindividual variability in gut microbial metabolism of (poly)phenols has led to the concept of (poly)phenol metabotypes, which classify individuals based on their ability to convert specific (poly)phenols into their corresponding gut microbial metabolites. However, the definition of metabotypes remains debated, with some considering it a qualitative classification (e.g., distinguishing metabolite producers from nonproducers), while others propose a quantitative approach, viewing metabotyping as a graded marker of metabolic capacity to predict individual responses to dietary (poly)phenols [25^▪^]. Regardless of the classification approach, only a few (poly)phenol metabotypes – particularly those involved in isoflavone, urolithin, and resveratrol metabolism – have been well characterized. These metabotypes are associated with distinct microbial ecologies and metabolite profiles; however, no definitive evidence has yet established a clear link between specific metabotypes and favourable health outcomes or responses to dietary interventions. Understanding these variations is essential, as they may help explain individual differences in responses to (poly)phenol consumption and can serve as potential biomarkers for precision nutrition strategies.

Among the most studied (poly)phenol metabotypes are those related to the isoflavone daidzein, sourced from soy products and red clover, such as equol producers (EP) and nonproducers (ENP), and O-desmethylangolensin (ODMA) producers and nonproducers. Findings from observational studies and RCTs on the relevance of equol and ODMA metabotypes for human health have been inconsistent, which we argue may be partly due to methodological differences in defining metabotypes, such as for example variations in producer vs. nonproducer cut-offs influenced by instrument detection limits [25^▪^]. A recent study introduces another possibility: the existence of a more complex cluster of isoflavone metabotypes [26^▪▪^]. In a group of postmenopausal women, five distinct clusters were identified based on their ability to metabolize daidzein into equol and ODMA, and genistein into 4-ethylphenol. Cluster 1 and Cluster 2 both produced equol but differed in genistein metabolism, with Cluster 1 being a “strong daidzein but low genistein” metabolizer and Cluster 2 efficiently metabolizing both isoflavones (“entire isoflavone” metabolizer). Cluster 3 individuals were “strong genistein” metabolizers, producing high levels of 4-ethylphenol but low levels of equol. Cluster 4 represented “poor isoflavone metabolizers,” while Cluster 5 had high levels of intermediate dihydro-metabolites but lacked the ability to convert them into equol, ODMA, or 4-ethylphenol (“interrupted isoflavone metabolizer”). These findings highlight the complexity of (poly)phenol gut microbial metabolism and suggest that further untargeted metabolomic studies are needed to reach a more refined classification of metabotypes which is necessary to clarify their health implications.

Urolithin metabotypes A, B, and 0 (UMA, UMB, and UM0) represent distinct metabolic responses to ellagitannins and ellagic acid, found in berries, pomegranates, and walnuts. Individuals with UMA exclusively produces urolithin A, while UMB individuals produce urolithin A, urolithin B, and isourolithin A, revealing a broader metabolic capacity. In contrast, those classified as UM0 exhibit no detectable production of urolithins. Variability in detection methods partially explains differences in UM0 prevalence across populations. Evidence suggests a higher proportion of UMB in older adults, indicating a potential link between aging and metabotype B [27]. Beyond age, urolithin metabotype distribution may be influenced by ethnicity, sex, and health status, however little is currently known. Notably, UMA is associated with a better cardiometabolic profile, while UMB, with its greater microbial adaptability, may benefit more from (poly)phenol-rich interventions due to its ability to engage with diverse microbial pathways [28^▪▪^]. A recent RCT suggest urolithin A production influences lipid metabolism [29], while urolithin A consumption improved gut barrier integrity in an animal model [30]. However, further research is essential to uncover the underlying mechanisms of these relationships, especially regarding how dietary modifications can convert nonproducers into metabolite producers, potentially improving health outcomes across diverse populations. Another promising strategy is the administration of probiotic bacteria able to metabolize (poly)phenols to nonproducer individuals. This approach has been successfully tested in an animal study, where two bacterial consortia colonized the gut of nonurolithin-producing (UM0) Wistar rats, enabling urolithin production and mimicking UMA and UMB metabotypes. After 4 weeks of oral administration, the urolithin producing bacteria established in the gut, effectively transferred urolithin production capability, and were well tolerated, with no major alterations in gut microbiota except for a reduction in *Streptococcus*[31^▪▪^].

More recently, after consumption of grape extract, two resveratrol-related metabotypes – lunularin producers (LP) and nonproducers (LNP) – have been identified, with LP individuals exhibiting greater microbial diversity and enriched functional pathways [32^▪▪^]. The gut microbiota associated to these metabotypes remains unclear, as well as their implications for human health. Xanthohumol is a prenylated flavonoid found primarily in hops, a key ingredient in beer, and to a lesser extent in hop-derived supplements and certain herbal products. In a randomized controlled trial in healthy individuals, xanthohumol supplementation for 8 weeks induced mild, enterotype-dependent changes in gut microbiota, notably affecting the Prevotella enterotype and reducing microbial-derived bile acid metabolism. While no specific metabotypes were proposed, significant interindividual variability was observed in xanthohumol gut microbial metabolism [33].

Several attempts have been made to identify flavan-3-ol metabotypes, but the complexity of their metabolism and the nonspecificity of end products present significant challenges. A human intervention study on cranberry consumption identified three distinct urinary metabotypes, primarily driven by colonic metabolism and characterized by differences in phenyl-γ-valerolactones, hydroxycinnamic acids, and phenylpropanoic acids, with 5-(3’,4’-dihydroxyphenyl)-γ-valerolactone derivatives emerging as key markers [34^▪^]. Another approach using faecal batch fermentations of healthy donors inoculated with (−)-epicatechin identified fast and slow converters, as well as high producers of 1-(3′-hydroxyphenyl)-3-(2″,4″,6″-trihydroxyphenyl)-propan-2-ol and 5-(3′,4′-dihydroxyphenyl)-γ-valerolactone. Fast conversion was associated with short chain fatty acid producing bacteria (Faecalibacterium and Bacteroides) and elevated levels of acetate, propionate, butyrate, and valerate, while Lachnospiraceae UCG-010 and Adlercreutzia equolifaciens were linked to specific metabolite transformations [35^▪^]. Recent findings using the dynamic M-SHIME model demonstrated that incubating (+)-catechin with microbiota from fast and slow converters resulted in distinct metabolic patterns, varying by colon region, with upstream metabolites in the proximal colon and downstream metabolites in the distal colon. Eggerthella and Flavonifractor, key catechin-metabolizing bacteria, were more abundant in the distal colon and in slow converters, emphasizing their role in catechin metabolism [36^▪^].

Given the widespread presence of diverse (poly)phenols in the diet, which are typically consumed together, a key yet understudied aspect is the interaction between different (poly)phenol metabotypes and their combined effects on metabolism and health. Recent findings suggest distinct associations between lunularin production (LP and LNP), equol (EP and ENP) and urolithin metabotypes (UM0, UMA and UMB). The most common metabotype clusters include LP + UMB + ENP (22.7%), LP + UMA + ENP (21.3%), LP + UMA + EP (16.7%), and LP + UMB + EP (16%), highlighting potential microbial-driven clustering. Notably, gut microbiome composition appears to be the key mediator, while sex, BMI, and age do not significantly differ across these clusters [28^▪▪^]. This evidence on metabotype clustering highlights the need to investigate additional (poly)phenol classes to elucidate metabolic pathways and common metabolic signatures.

## SYNERGISTIC POTENTIAL OF (POLY)PHENOLS AND PROBIOTICS: A SYNBIOTIC APPROACH

Recent advances in synbiotic strategies are paving the way for (poly)phenol-based interventions, where prebiotics and probiotics work synergistically to enhance gut and host health. In this context, (poly)phenols serve a dual role: as substrates promoting probiotic engraftment and as modulators of microbial metabolism [37^▪^]. For example, green tea (poly)phenols in combination with probiotics, including *Lactobacillus*, is a promising candidate for promoting growth of beneficial microbes and immune defence mechanisms [38^▪^]. Dietary modifications incorporating (poly)phenol-rich fermented foods, such as kimchi and natto, have also emerged as promising interventions targeting mental health [39^▪^], while kombucha has been shown to enhance probiotic delivery with yeast and proliferate beneficial microbial species like *Gluconacetobacter xylinu*[40^▪▪^].

Fortified and fermented foods further illustrate the synergy between (poly)phenols and probiotics. Yogurt enriched with olive (poly)phenol has reported to promote growth of lactic acid bacteria *in vitro*, and has reduced low-density lipoprotein (LDL) levels in clinical trials, underscoring the potential for combining (poly)phenols with probiotics to enhance metabolic health [41]. Similarly, novel fermentation techniques have been explored to increase the bioactivity of (poly)phenols. For instance, probiotic fermentation of tea leaves enhances the antibacterial properties of tea phenolic compounds and their metabolites, which promotes the production of beneficial bacteria species, including Bifidobacterium and Lactobacillus, thereby providing gut homeostasis benefits [42]. A recent in vitro model has confirmed that probiotic fermentation of pineapple flavonoids can also increase SCFA-producing microbes and improve intestinal gut microbial balance of faecal microbiota transplant donors, highlighting fermentation as a strategy to amplify the therapeutic potential of dietary (poly)phenols [43].

## (POLY)PHENOL METABOLITES AS POSTBIOTICS

Beyond synbiotics, postbiotics are gaining recognition as key modulators of gut and systemic health. Postbiotics have been defined as a “preparation of inanimate microorganisms and/or their components that confers a health benefit on the host” [44]. This definition includes a diverse range of microbial-derived components, such as metabolites, cell wall fragments, and inactivated microbial cells, which exert beneficial effects such as immune modulation, reinforcement of gut barrier integrity, and short-chain fatty acid (SCFA) production. Unlike probiotics, postbiotics do not require colonization, offering greater stability, safety, and consistency in therapeutic applications, particularly for individuals with gut dysbiosis [45^▪^].

(Poly)phenol metabolites, produced through gut microbial biotransformation, can function as postbiotics by modulating gut microbiota composition and exerting anti-inflammatory and metabolic effects. For instance, urolithin A – ellagitannin-derived gut microbial metabolites – have demonstrated anti-inflammatory properties and mitochondrial benefits on muscle performance [46], while dihydroresveratrol, a gut-derived metabolite of resveratrol, exhibits enhanced bioavailability and bioactivity [47]. Similarly, consumption of microbial catabolites of flavonoids, such as equol (from daidzein) and 3,4-dihydroxyphenylacetic acid (from quercetin), have been linked to improved cardiovascular and intestinal health outcomes [48].

These findings highlight postbiotics, including (poly)phenol-derived metabolites, as promising candidates for precision nutrition approaches targeting gut microbiota, metabolic health, and chronic disease prevention.

## CONCLUSION

While significant progress has been made in understanding interindividual variability in (poly)phenol gut microbial metabolism, many gaps remain. For most (poly)phenols, the link between gut microbial metabolizing capacity and associated gut microbial signatures remains unclear. Limited evidence suggests that gut microbiota composition, genetic factors, diet, and lifestyle shape microbial enzymatic capacity and metabolic pathways, yet the key determinants of metabotypes are not well established. Crucially, the relationship between metabotypes and individual responses to (poly)phenol interventions remains largely unexplored, with minimal evidence from RCTs. A major challenge is that only a few (poly)phenol subclasses have well characterized, traceable metabolites, while most generate common, nonspecific metabolites from multiple dietary and nondietary sources. Furthermore, large-scale studies in representative populations are needed to establish robust clustering of different metabotypes. Advancing the field requires robust clinical methodologies, including sensitive biochemical analyses and standardized (poly)phenol challenges, to ensure accurate metabotype stratification, particularly in identifying nonproducers. Future research should prioritize characterizing metabolic signatures across diverse populations to establish metabotyping as a cornerstone of precision nutrition interventions. Integrating machine learning and multiomics approaches (metabolomics, microbiomics, genomics) will be key to developing predictive models that elucidate the complex interplay between diet, gut microbiota, and health outcomes, ultimately guiding more effective and personalized dietary strategies.

## Acknowledgements

*None.*

### Financial support and sponsorship

*None.*

### Conflicts of interest

*There are no conflicts of interest.*

## REFERENCES AND RECOMMENDED READING

Papers of particular interest, published within the annual period of review, have been highlighted as:

▪ of special interest
▪▪ of outstanding interest
